# Genome Sequence of the Cluster EF Bacteriophage TinyMiny, Isolated using *Microbacterium foliorum*

**DOI:** 10.17912/micropub.biology.001488

**Published:** 2025-05-29

**Authors:** Iain Duffy, Juan Jimenez, Luis Gomez, Alexa Keeler, Aisha Ambrose, Moises Guadarrama, Hailey Kerns, Rachael Martin, Jade Moorehead, Arrianne Orgill, Brianna Reiber, Aisha Severe, Leslie Sivley, Genesis Valentin, John Duncan

**Affiliations:** 1 Natural Sciences, Saint Leo University, Saint Leo, Florida, United States

## Abstract

The bacteriophage TinyMiny was isolated from a soil sample collected on the campus of the University of Maryland Baltimore County, in Baltimore, Maryland, using the bacterium *Microbacterium foliorum* NRRL B-24224. TinyMiny is presumed to be a lytic bacteriophage, consisting of a double-stranded DNA genome 57,042 base pairs in length and encoding 86 genes. Based on gene content similarity to actinobacteriophages, TinyMiny is assigned to the EF cluster.

**Figure 1. Virion Morphology of bacteriophage TinyMiny:**
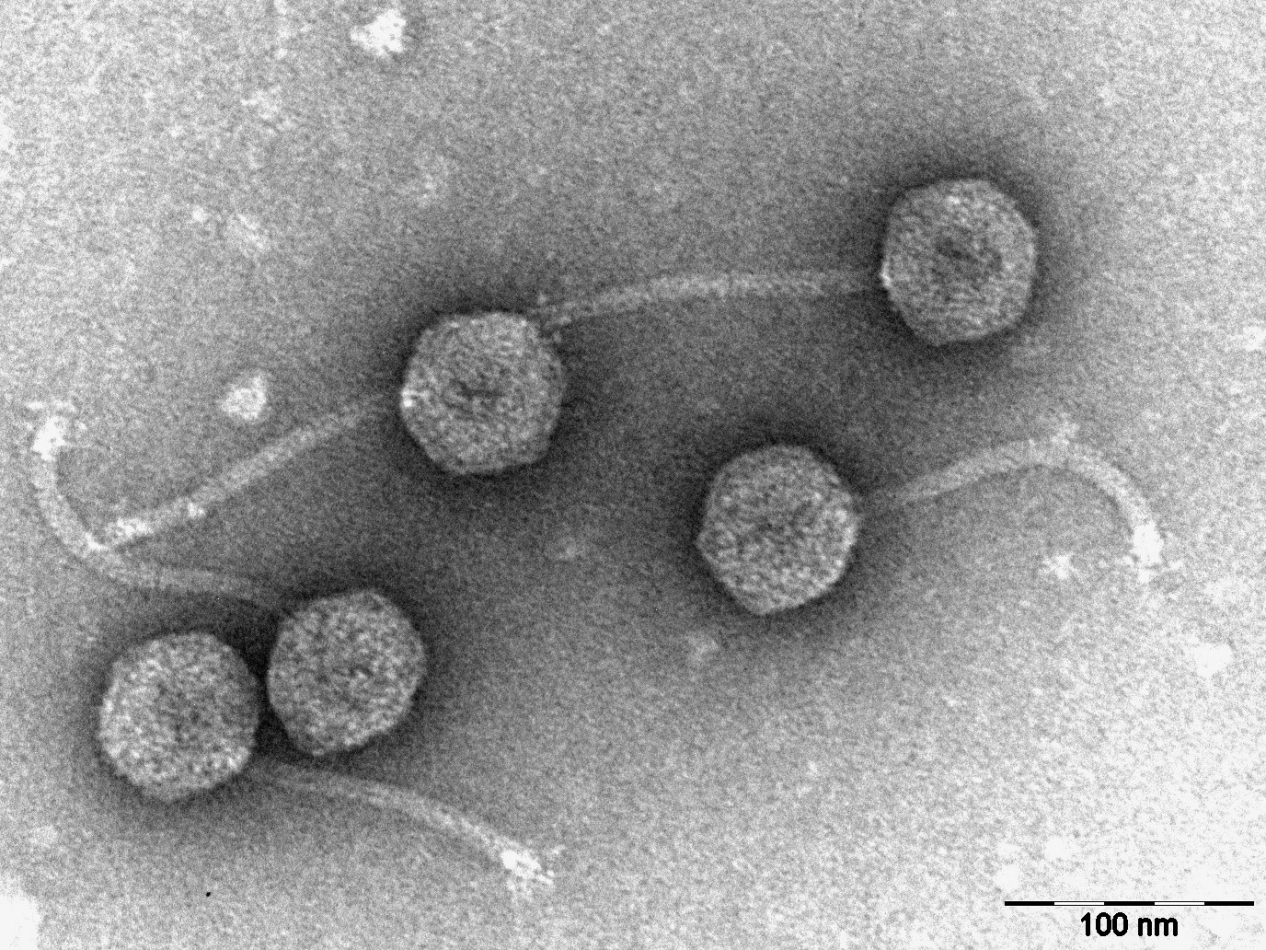
A negatively stained (1% uranyl acetate) transmission electron micrograph of TinyMiny reveals a siphoviral morphology with an icosahedral capsid and a flexible tail. Scale bar is 100 nm.

## Description

Bacteriophage TinyMiny was isolated as part of the effort by the Science Education Alliance-Phage Hunters Advancing Genomic and Evolutionary Science (SEA-PHAGES) program, supported by the Howard Hughes Medical Institute, to uncover the genetic diversity of actinobacteriophages, including those that infect *Microbacterium foliorum* ( *M. foliorum* ) (Jordan et al., 2014; Hatfull GF. 2020; Jacobs-Sera et al., 2020) . Using standardized protocols, a soil sample was collected from under a layer of mulch beneath a bush (GPS coordinates: 39.255319 N, 76.708294 W) when the ambient temperature was 25°C (Zorawik et al., 2024). The sample was suspended in peptone yeast calcium (PYCa) liquid medium and shaken at 220 rpm for 2hrs at 30 ^o^ C, following which, the sample was spun in a centrifuge at 2,000 rpm for 10 minutes and the supernatant filtered through a 0.22-µm pore filter. To this filtrate was added *M. foliorum* NRRL B-24224 and the suspension further incubated with shaking at 30°C for 24 hours to enrich for phages capable of infection of *M. foliorum* . Following incubation, the resulting culture was filtered, and the filtrate plated in *M.* *foliorum* PYCa top agar overlays and incubated at 30 ^o^ C. Bacteriophage TinyMiny was purified via two rounds of plating, after which a lysate was prepared. TinyMiny formed plaques that were <1mm at 24 hours, which enlarged to approximately 1mm at 48 hours. Negative staining (1% uranyl acetate) transmission electron microscopy revealed TinyMiny to have a siphoviral morphology with an icosahedral capsid 62-65 nm in diameter and a tail 127-132 nm in length (n=5) (Figure 1).

TinyMiny DNA was extracted from a lysate using the Wizard DNA Clean-Up Kit (Promega, Madison, WI). This DNA was then prepared for sequencing at Pittsburgh University using the Ultra II Library Kit (NEB, Ipswich, MA) and sequenced using an Illumina MiSeq (v3 reagents), yielding 316,038 150-base single-end reads that provided 815-fold coverage of the genome when assembled using Newbler v2.9 (Miller et al., 2010; Russell 2018). The assembled genome was checked for completeness and genome termini using Consed v29 (Gordon et al., 2013). TinyMiny has a circularly permuted genome of 57,042 base pairs, with a GC content of 62.7%.

Annotation of the TinyMiny genome was accomplished using DNA Master v5.23.6 (http://cobamide2.bio.pitt.edu/) (Pope et al., 2018), Glimmer v3.02 (Delcher et al., 1999), GeneMark v2.5p (Besemer and Borodovsky 2005), PECAAN v20211202 (http://blog.kbrinsgd.org/), Starterator v485 (https://seaphages.org/software/#Starterator), Blast searches (Altschul et al., 1990) against the NCBI non-redundant protein and Actinobacteriophage (https://phagesdb.org/) databases (Russell and Hatfull, 2017), HHPred v3.2 (searching against the PDB_mmCIF70, SCOPe70, Pfam-A v36, NCBI Conserved Domain database v3.19) (Zimmermann et al., 2018), Phamerator v.37 (Cresawn et al., 2011), DeepTMHMM v1.0.24 (Hallgren et al., 2022), Aragorn v1.2.41 (Laslett and Canback, 2004) and tRNAscan-SE 2.0 (Chan and Lowe 2019). All software used in this annotation utilized the default settings.

The annotation process resulted in 86 genes being predicted for TinyMiny, 27 of which could be assigned a putative function. No tRNAs were predicted. Based on gene content similarity of >35% to bacteriophages in the Actinobacteriophage database, TinyMiny was assigned to cluster EF (Gauthier et al., 2023). TinyMiny encodes 42 homologues that are shared across the EF cluster including a terminase, portal, major tail, major and minor capsid proteins, minor tail proteins, tail assembly chaperones, tape measure protein, DNA helicase, DNA primase, a RuvC-like resolvase, and two related alpha subunits of RNA polymerase III. The endolysin function identifiable in cluster EF phages belong to two different protein phamilies, with TinyMiny encoding the endolysin that is encoded by a minority (6 other) of EF phages (Cresawn et al., 2011). No immunity repressor, integrase, or DNA partitioning proteins could be identified in TinyMiny or any other cluster EF phage, suggesting they are unlikely to establish lysogeny.

**Nucleotide sequence accession numbers**

The GenBank Accession No. for TinyMiny is MZ681513 and Sequence Read Archive (SRA) No. SRX14485096 .
